# Spatial Mapping of Electrochemical Hole Injection into Supercrystals of Perovskite Nanocrystals

**DOI:** 10.1021/acs.nanolett.6c01326

**Published:** 2026-07-14

**Authors:** Theresa Hettiger, Jonas L. Hiller, Richard Hodak, Martin Eberle, Alfred J. Meixner, Marcus Scheele

**Affiliations:** Institute of Physical and Theoretical Chemistry, Auf der Morgenstelle 18, 72076 Tübingen, Germany

**Keywords:** lead halide perovskites, nanocrystals, superlattices, spectroelectrochemistry

## Abstract

Lead halide perovskite nanocrystals are of interest for application as an emissive layer in light-emitting diodes (LEDs) due to their high photoluminescence quantum yield, their color tunability, and their defect tolerance. For the application in LEDs, charges need to be easily transferred from the charge transport layers to the nanocrystal core without compromising the optoelectronic properties. While charging of nanocrystals can be studied by spectroelectrochemistry (SEC) in general, in this work, we combine SEC with diffraction-limited optical resolution. This allows us to study hole injection into self-assembled supercrystals of CsPbBr_3_ nanocrystals and determine the spatially dependent potential for this action. We find an overall brightening of the photoluminescence, which is gradually shifting from the edges to the supercrystal center, and a generally greater resistance toward electrochemical degradation in the supercrystals.

## Graphical abstract

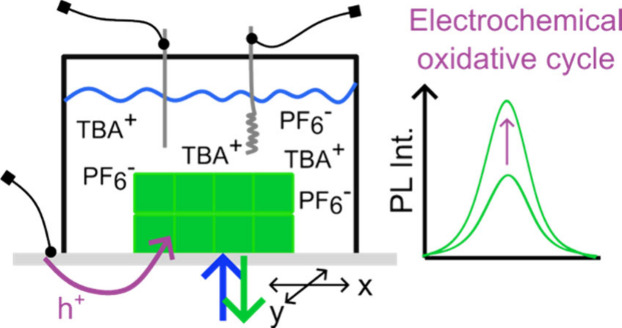

Thin films of lead halide perovskite nanocrystals (NCs) are promising for application in light-emitting diodes (LEDs)^1–3^ since they show high photoluminescence (PL) quantum yield,^4–6^ narrow emission line width,^7^ and a wide color gamut enabled by anion exchange.^8,9^ In addition to these properties, self-assembly of lead halide perovskite NCs into supercrystals (SCs)^10,11^ gives rise to collective coupling effects, such as superfluorescence,^12^ minibands,^10,13^ and amplified spontaneous emission.^14^ The high PL intensities and narrow line widths resulting from these phenomena have sparked the idea to build micro-LEDs from single lead halide perovskite SCs.^15,16^ However, to realize such devices, several obstacles that are specific to SCs need to be overcome. In particular, charge injection and transport through the SC are not trivial since typical SCs are much thicker (>1 μm) than most active layers in NC-based LEDs (<100 nm). In addition, the poor long-term stability of lead halide perovskite NCs, especially under bias, remains a challenge.^17–19^

We and others have recently shown that spectroelectrochemistry (SEC) is an ideal tool to address these questions.^20–23^ In particular, SEC is a powerful method to study band alignment in heterostructures,^24,25^ charge injection potentials,^26^ and surface passivation/trap formation on the surface.^27,28^ Due to the high sensitivity of SEC, it is even possible to investigate single NCs and determine the number of transferred electrons.^29–31^

However, SEC of individual lead halide perovskite SCs has not yet been reported. Most likely, this is because of their dynamic surface chemistry^32^ and halide mobility,^33^ which leads to irreversible electrochemical oxidation and limits the time window for performing such measurements.^33–36^ Nonetheless, hole injection into thin films of CsPbBr_3_ NCs has recently been demonstrated.^23,33,37^

In this work, we successfully measure the PL under SEC control of individual CsPbBr_3_ SCs with diffraction-limited spatial resolution in the electrochemical window of hole injection. To this end, we alter the surface of the NCs to achieve facilitated charge injection and high stability in polar solvents. For SCs self-assembled from such modified NCs, we observe hole injection followed by an increase in PL, starting from the edges toward the center of the SCs. Spectral mapping after SEC reveals an overall increase in the PL intensity of the SCs, which highlights their robustness against degradation by electrochemical hole injection.

For the SEC experiments in this work, the NCs need to allow for relatively fast charging rates and high stability against polar solvents, such as propylene carbonate (PC),^38^ which is typically not afforded by as-synthesized lead halide perovskite NCs in view of their insulating and labile oleic acid (OA)/oleylamine (OAm) ligand shell. In this work, we consider both factors in the pretreatment of the NCs. Therefore, we perform a ligand exchange in solution to a partial lecithin coverage. As a zwitterionic ligand, it is described to bind more tightly to the NC surface and shows higher stability against polar solvents during washing procedures.^39,40^ Afterward, we precipitate the NCs with acetone as antisolvent to remove excess ligands. We monitor the effect of this pretreatment on the surface chemistry of the NCs by quantitative NMR spectroscopy. From SI, Figure S1, we can deduce a surface coverage of 561 OA/OAm and 50 lecithin molecules per NC. This deviation from the theoretical full-surface coverage of 953 ligands per NC, assuming 2.9 ligands/nm^2^,^32^ facilitates charge injection through free surface sites. The sample preparation process is schematically depicted in Figure 1a.

**Figure 1:**
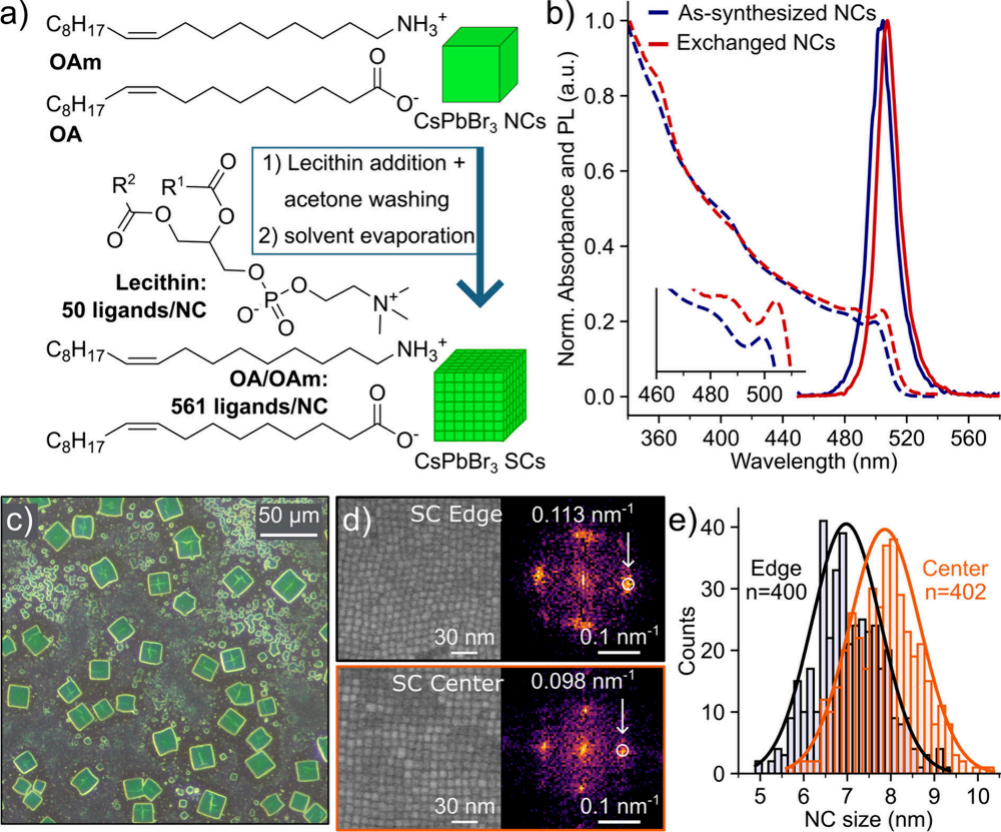
Characterization of CsPbBr_3_ NCs and their self-assembled SCs. **a)** Scheme of the NC postsynthetic treatment. **b)** Absorbance (dashed lines) and PL spectra (solid lines) of as-synthesized NCs (blue) and NCs after partial ligand exchange (red). **c)** Dark field optical micrograph of SCs, self-assembled from the exchanged NCs by slow evaporation of the solvent on FTO substrates. **d)** High-resolution SEM characterization of an SC with comparison of NC sizes on the edges of the SC (black) to the center of the SC (orange). Size determination by FFT analysis of SEM image (in **d**) and **e)** measuring edge lengths of *n* NCs and fitting to a normal distribution (right).

Figure 1b displays the steady-state optical absorbance and PL spectra of the CsPbBr_3_ NCs as obtained from synthesis and after successful ligand exchange in solution. Following the ligand exchange, a red-shift of approximately 4 nm is observed. Specifically, the first excitonic transition (see inset) shifts from 499 to 504 nm, while the PL maximum shifts from 503 to 507 nm. This red-shift suggests a larger NC size after the exchange, which we corroborate by electron microscopy to find an increase of the average NC diameter from 7.4 ± 0.8 nm to 8.2 ± 0.9 nm (SI, Figure S2). The increase in size and PL maximum can be attributed to Ostwald ripening during the ligand exchange by antisolvent precipitation.^41–44^ Additionally, the exchanged NCs exhibit more pronounced first and second excitonic transitions, along with a reduction in the PL full width at half-maximum from 17.5 to 16.3 nm.

By slow evaporation of the solvent, square SCs form readily from the exchanged solutions on conductive and transparent fluorine-doped tin oxide (FTO) substrates, as shown in Figure 1c. These SCs range in size from approximately 5 × 5 μm^2^ to 30 × 30 μm^2^ (SI, Figure S3). Figure 1d shows high-resolution SEM images acquired from the edge and the center of a representative SC and their respective fast Fourier transforms (FFTs). As evident from the SEM images and the azimuthal broadening of the first-order FFT signals, the order of the SC lattice is more pronounced in the center. From the reciprocal of the indicated distance to the zero-order frequencies, we determine an average NC center-to-center distance (NC + interdigitated ligand shell) of 8.8 and 10.2 nm at the SC edge and center, respectively. We compare these values with the manually measured edge lengths of the inorganic NC cores in the histogram in Figure 1e, yielding average edge lengths of approximately 7 and 8 nm at the SC edge and center, respectively. The difference accounts for the ligand shell with 1.8 nm at the edges and 2.2 nm in the SC center.

Figure 2a depicts a sketch of the cell used for SEC PL experiments. The cell creates a solvent-tight seal around the central part of an FTO working electrode (WE) in a volume containing a Ag wire pseudoreference electrode (pRE) and a Pt coil counter electrode (CE). The cell, with all three electrodes connected to a potentiostat, can be placed on top of an inverted confocal laser-stage scanning microscope to track the response of the spatially resolved PL to polarization changes of the working electrode. A detailed description of the cell and measurement setup is given in the Materials and Methods section of the SI. All potentials stated in this work are referenced to the half-wave potential of the ferrocene/ferrocenium redox couple recorded in the SEC cell (SI, Figure S6, vs Fc/Fc^+^, which will be omitted in the following).

**Figure 2:**
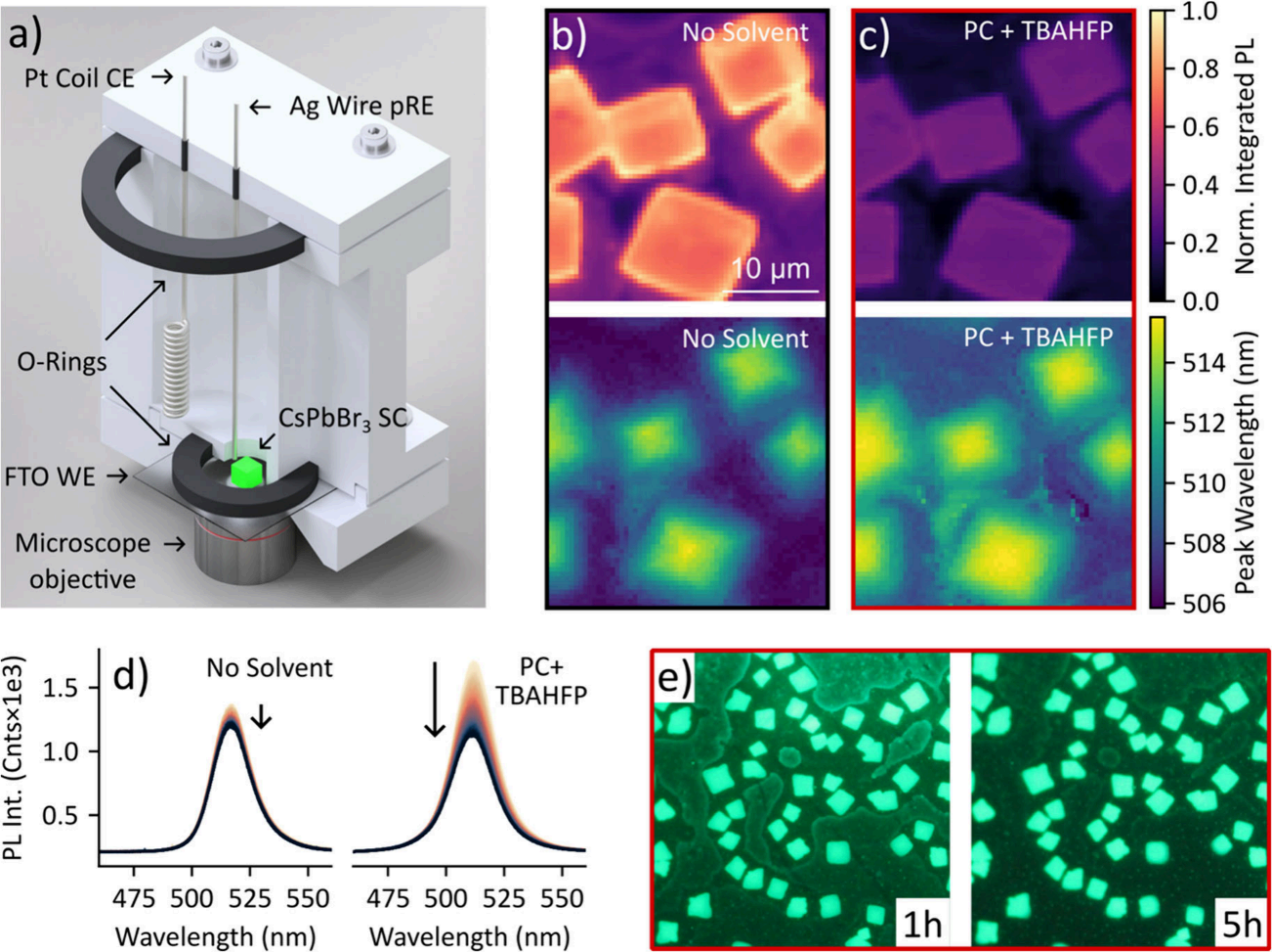
Stability of CsPbBr_3_ SCs against the electrolyte solution. **a)** Sketch showing a sectioned view of the cell used for emission SEC experiments, where CE = counter electrode, pRE = pseudoreference electrode, WE = working electrode. **b)**, **c)** Corresponding maps of intensity (top) and peak wavelength (bottom) of a sample of CsPbBr_3_ SCs on an FTO WE before and after the addition of ES to the cell, respectively. **d)** PL intensity bleaching of SCs under continued laser irradiation over 300 s both in the absence (left) and in the presence (right) of ES. **e)** PL micrographs of a sample of CsPbBr_3_ SCs on a FTO WE after exposure to the ES for 1 h (left) and 5 h (right) excited by a low-power UV LED.

Figure 2b displays maps of the PL intensity (top) and the peak wavelength (bottom) emitted by CsPbBr_3_ SCs on the FTO working electrode. We find that the brightly emitting SCs are surrounded by a weaker-emitting thin-film region of NCs. With 506 nm, the PL from this film exhibits roughly the same peak wavelength as the NCs in solution. In contrast, the PL of the SCs is red-shifted and displays an increase in peak wavelength along a radial gradient from around 508 nm at the SC edges up to around 514 nm in the SC center. We attribute the overall red-shift of the NCs assembled in the SC to coupling/mini-band formation,^10^ while the effect in red-shifted emission toward the SC center is presumably caused by the increase in NC size (see Figure 1e).

The corresponding maps of the same sample area after the addition of the electrolyte solution (ES), composed of tetrabutylammonium hexafluorophosphate (TBAHFP) in PC, recorded using identical acquisition parameters, are depicted in Figure 2c. We observe a drop in the overall PL intensity of around 50–60%, and no significant shift of the peak wavelength (SI, Figure S7).

For SEC experiments, the stability of the employed CsPbBr_3_ SCs against both degradation due to the polar ES and photobleaching due to the focused laser excitation needs to be considered. We argue that we can rule out degradation of the NCs within the time frame of the measurements (approximately 75 min for each combined intensity plus spectral map acquisition), as this would lead to a blue-shifted emission due to NC shrinkage.^45–47^ Contrary to this, we observe a slight red-shifting of the PL across the entire probed area on the order of 1 nm, which could be attributed to changes in the dielectric environment due to the presence of the ES. The evolution of the PL intensity recorded from SCs with and without the presence of the ES under 300 s of continuous focused laser excitation is depicted in Figure 2d. Although the exact rate of photobleaching varies between individual SCs even at a constant excitation density, we generally observe more pronounced photobleaching in the presence of the ES, which we attribute to the polar environment promoting ligand detachment. Figure 2e displays photoluminescence micrographs recorded from the same area of CsPbBr_3_ SCs on a FTO WE after 1 and 5 h of continuous exposure to the ES. The SCs retain their morphology and exhibit bright emission even after 5 h.

We conclude that while focused laser excitation induces photobleaching, SCs assembled from NCs with partial lecithin ligand exchange are sufficiently stable in the chosen ES to enable spectroelectrochemical experiments.

In the next step, we performed SEC on the self-assembled SCs under 405 nm laser excitation. Therefore, we record an intensity map of a specific region, and we choose 15 equally distributed positions across the SC, indicated by numbers. In Figure 3a, the positions and scan directions are displayed. Before the application of a potential, we recorded single spectra at each position as a reference PL_0_. The spectra are displayed in Figure 3b and show a red-shift of 3 nm of the PL peak wavelength to the center comparable to the data in Figure 2c.

**Figure 3:**
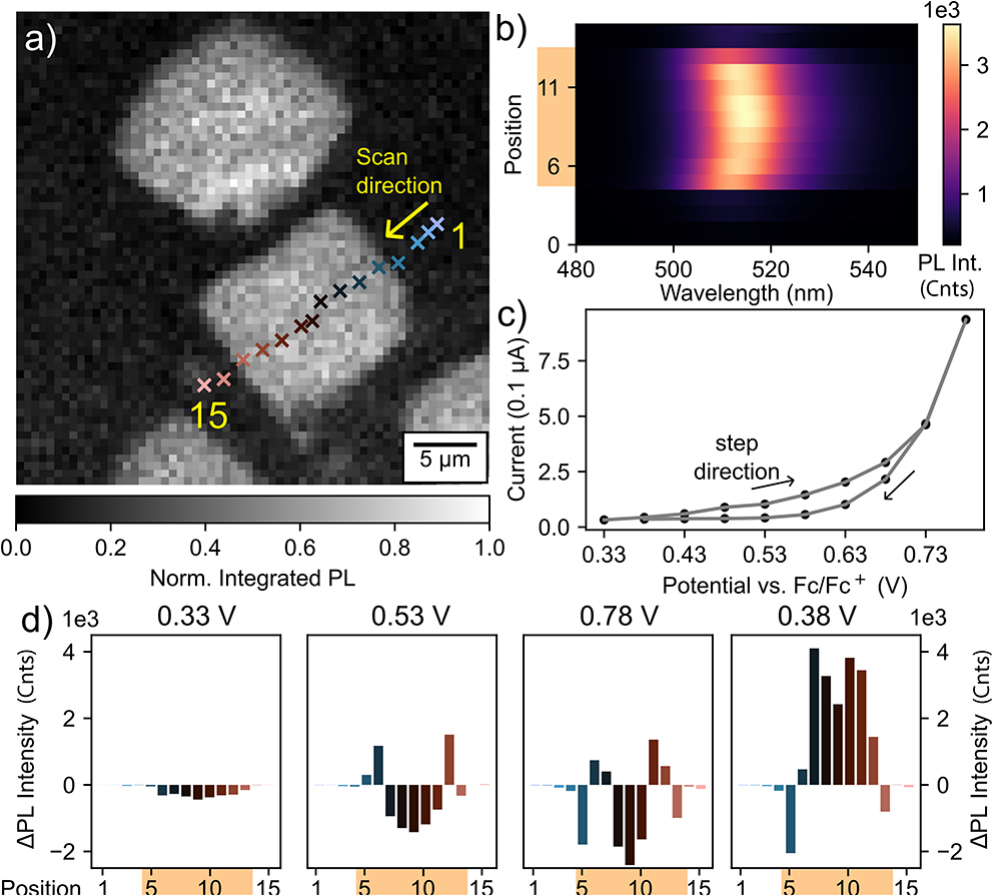
SEC PL on CsPbBr_3_ SC. **a)** Integrated intensity map of SCs on FTO with indicated scan direction and positions, where single spectra during SEC were acquired. **b)** PL spectra PL_0_ before SEC recorded at the specific positions. **c)** Recorded current during staircase voltammetry in the oxidative scan direction with arrows indicating the forward and backward scan. **d)** Change in PL maximum intensity (ΔPL = PL_E_ – PL_0_) at each marked position from 1 (on the left) to 15 (on the right) during the application of a potential at E = 0.33, 0.53, and 0.78 V in the forward scan and 0.38 V in the reversed scan direction. All spectra corresponding to positions in the SC are shaded in red.

As an electrochemical method, we choose staircase voltammetry (SCV) since each potential step can be kept for a longer period of time (210 s). This time period allows us to record PL spectra at each spatial position, after which we increased the potential by 0.05 V. The procedure for recording single PL spectra is then repeated. To focus on electrochemical hole injection, we perform a forward and a reverse scan within the limits of 0.33–0.78 V, since electrochemical hole injection is expected at roughly 0.5 V from previous reports.^23,33^ Furthermore, we anticipate irreversible bromide oxidation in solution to start at 0.78 V, which can lead to complete dissolution of the SCs (SI, Figure S8 and Figure S9). The SCV in Figure 3c shows the recorded current during the last 53 s, which omits the capacitive current. We observe an exclusively positive current, that is, an oxidative current, in both scan directions. However, the magnitude is larger in the forward scan than that in the reverse scan. The oxidative current onset was observed at 0.43 V.

Fundamentally, one would expect a decay in PL intensity as holes are injected into the VB, which is why we monitor any change in PL intensity (ΔPL) with respect to the PL intensity without applied potential (PL_0_) in Figure 3d. The ΔPL of the first potential (0.33 V), in the middle of the oxidative scan (0.53 V), the maximum potential (0.73 V), and the final recorded spectra (0.38 V) are shown. Full spectra for each potential are shown in Figure S10 as intensity maps. We find a PL bleach at every position for the first potential step at 0.33 V. Further increasing the potential (0.53 V) leads to an unexpected positive ΔPL on the edges of the SC (positions 5, 6, and 12), while ΔPL is still negative in the center. At the maximum potential (0.78 V), the negative ΔPL persists at the center positions (8, 9, and 10), while the positions further to the edges (6, 7 and 11, 12) exhibit positive ΔPL. At the very edges (positions 5 and 13) a bleach reappears. The last panel in Figure 3d summarizes the ΔPL during the reversed scan at 0.38 V, revealing a significant brightening of the PL for most positions in the SC except for the very edges. In another visualization of the same data, we track the position dependence of the potential required to invoke a positive ΔPL in the SC for the first time (SI, Figure S11). From this presentation, we deduce a shift of the onset potential of PL brightening by 0.1 V by stepping from position 5 to position 6 alone, that is, by scanning toward the center. We observe the same behavior for the opposite edge of the SC (Figure S11).

To verify our results, we perform similar measurements on another SC sample (Figures S14 and S15) but in a less oxidative potential window from 0.03 to 0.63 V. Qualitatively, we obtain the same results; however, this time only the edges brighten significantly while the center of the SC recovers to approximately the starting PL intensity when the scan direction is reversed (SI, Figure S12). This is consistent with the previous observation that PL brightening in the SC center requires larger (over)­potentials than on the edges. In line with this, we determine the potential at which the initial bleach on the forward scan begins between 0.18 V (outer edges) and 0.43 V (center) in Figure S16.

To determine if holes could be electrochemically injected by the applied potential, we perform SEC in absorbance with a thin film of the same surface-treated NCs and the same experimental SCV parameters. We find exclusively negative differential absorbance, with the greatest effect at 0.53 V (SI, Figure S20c). Since absorbance SEC is mostly unaffected by nonradiative trap states,^48,49^ we explain these observations with electrochemical hole injection starting between 0.38 and 0.43 V in the SCV (Figure 3c) and in the ΔPL plots (Figure 3d), which is in accordance with recent reports.^23,33,37^

For further validation, we recorded spectral maps before (Figure 4a) and after one SCV cycle with successful hole injection (SC1 in Figure 4b). Consistent with the results in Figure 3d, we observe an overall increase in PL intensity of the SCs after SEC. This applies likewise to SCs that were only briefly excited during the recording of the spectral maps (SC2 in Figure 4b).

**Figure 4:**
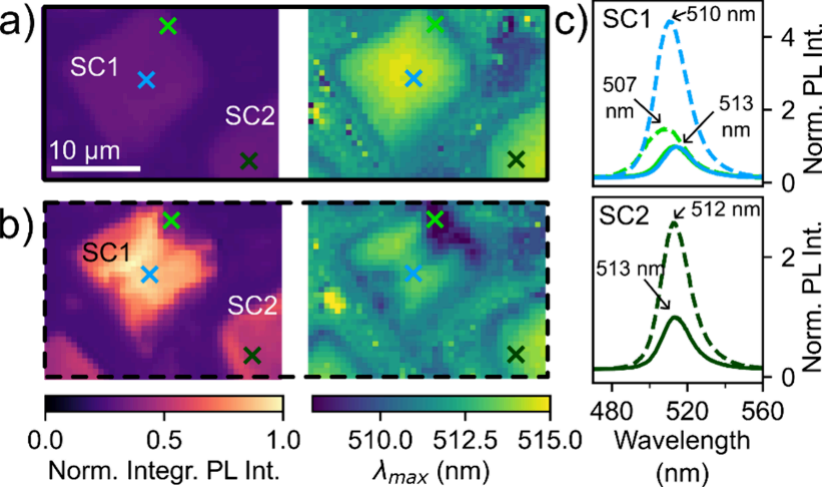
Spectral mapping of SC sample before and after SCV. **a)** Spectral map before SCV with normalized integrated intensity plot (on the left) and extracted PL maximum (λ_max_, on the right). **b)** Spectral map after SCV. **c)** Extracted single spectra from spectral maps before SCV (solid line) and after SCV (dashed line) normalized to the maximum intensity before SCV with marked maximum PL intensities. The positions of the spectra in the spectral maps are highlighted in panels a) and b).

In Figure 4c, we extract four individual spectra for SC1 before (solid line) and after SCV (dashed line) from positions at the edge (green) and the center (blue). For SC2, where SCV was conducted without simultaneous excitation, the corresponding spectra before (solid) and after (dashed) are shown for a single position. The peak PL wavelength (λ_max_) stays mostly constant in the center of the SCs. At the edges and along the line scanned by the laser, we observe occasional blue-shifting of λ_max_. The single spectra show a 2.6-fold PL intensity increase for SC2, while SC1 exhibits a 1.5-fold PL brightening on the edge (green) and 4.4-fold PL intensity increase at the center position (bright blue). We also observed PL brightening for another SC sample during a comparable SEC experiment (SI, Figure S22). The recorded spectral maps show that the SCs are resistant to degradation by electrochemical charge injection, and the charge injection is not limited by irreversible bromide oxidation (SI, Figure S8). Preliminary fluorescence lifetime measurements (Figure S23) after oxidative electrochemical cycling reveal that bright SCs exhibit longer average lifetimes (11 ns) than relatively dim SCs (2.9 ns). This indicates that electrochemically induced brightening may be linked to a depletion of (fast) nonradiative recombination.

To demonstrate the statistical relevance of our findings, we track the PL intensity changes during electrochemical cycling (0.33–0.73 V) of a larger sample area containing 22 SCs (Figure S24). We find PL brightening for over 90% of these SCs with a mean increase to (126 ± 22)% of the initial PL intensity.

In general, PL brightening is not anticipated since electrochemical hole injection would typically result in PL bleaching. We suggest that one possible explanation for the unexpected PL enhancement (during hole injection) is oxidative defect passivation, similar to the previously described “photobrightening” of lead halide perovskites.^50,51^ Briefly, it is reasonable to assume that exposure to the polar ES and/or the oxidative potential facilitates the formation of Br^–^ vacancies on the NC surface. Such vacancies have been shown to facilitate the reduction of Pb^2+^ to Pb^0^, which introduces trap states and a reduced PL.^52,53^ The following “brightening” due to trap state passivation is reported to occur by the formation of PbO on the surface^53^ or due to electrochemical removal of electron-rich hole traps.^54^ While these effects have been reported previously for bulk lead halide perovskites,^53^ they were found to be weak, at a level of a few percent, in NC films.^54^ The hypothesis of surface defect passivation is supported by the reduced PL intensity after the addition of ES (Figure S7) and the recovery of PL intensity of the overall SC sample after electrochemical oxidation (Figure 4c).

In conclusion, we have performed spatially resolved PL SEC on perovskite SCs self-assembled from CsPbBr_3_ NCs. Our results highlight their stability toward the presence of polar electrolyte solution and electrochemical hole injection. Under mild oxidative cycling, the PL intensity is not only preserved throughout the SC but even enhanced, which we attribute to electrochemical filling of hole traps. Our work shows that SCs are robust to electrochemical degradation, an advantage that can help achieve long-term stability of SCs in LEDs under electrical bias. We note a recent work on CsPbBr_3_ NC superlattice LEDs with greatly enhanced operational lifetimes that supports our conclusion.^16^

## Supplementary Material



## Notes

Experimental details and methods; ^1^H NMR spectra; SEM characterization before crystallization; SC characterization; SEC PL cell; single spectra from spectral maps in Figure 2; SEC PL with decomposition of SCs caused by bromide oxidation; extended data evaluation PL SEC Figure 3; PL SEC scan up to 0.63 V; absorbance SEC; time-resolved PL; PL brightening after SEC; statistics on PL brightening and edge brightening (PDF)
