# Supplementary material for: Spatial Mapping of Electrochemical Hole Injection into Supercrystals of Perovskite Nanocrystals
Source: Nano Lett. 2026 Jul 14;26(29):9403–9. doi: 10.1021/acs.nanolett.6c01326 (PMC13430685; doi:10.1021/acs.nanolett.6c01326)
Supplement: Supplementary file 1 [file nl6c01326_si_001.pdf]

Supporting Information:

Spatial Mapping of Electrochemical Hole-Injection into Supercrystals of Perovskite Nanocrystals

Theresa Hettiger[1], Jonas L. Hiller[1], Richard Hodak[1], Martin Eberle[1], Alfred J. Meixner[1] and Marcus Scheele[1,*]

[1]Institute of Physical and Theoretical Chemistry, Auf der Morgenstelle 18, 72076 Tübingen, Germany

*Corresponding author

marcus.scheele@uni-tuebingen.de

Content

S1. Material and methods.
S2. Qualitative and quantitative ^{1}H NMR spectroscopy.
S3. NC size before and after ligand exchange.
S4. SC characterization.
S5. PL spectra extracted from spectral map in Figure 2b+c.
S6. SEC PL cell and referencing by Fc/Fc^{+} redox couple.
S7. SEC PL including bromide oxidation from solution.
S8. Evaluation of peak PL intensity from SEC PL in Figure 3.
S9. SCV up to 0.63 V.
S10. Absorbance SEC on NC thin-film.
S11. Spectral mapping of 2nd example.
S12. Time-resolved PL after electrochemical hole injection.
S13. Statistics on PL enhancement after conducting a SCV scan.
S14. Edge brightening.

S1. Material and methods

Materials.
caesium carbonate (Cs_2CO_3, 99.92 % trace-metal basis, Acros Organics), 1-octadecene (ODE, 90 %, technical grade, Sigma Aldrich), oleyl amine (90 %, technical grade, Sigma Aldrich), oleic acid (OA, 90 %, technical grade, Sigma Aldrich), lead(II) bromide ($PbBr_2$, 99 %, Acros Organics), L-α lecithin (min. ≥ 97 % phospholipids, Thermo Scientific), FTO (1.1 mm thickness, ~20 Ω/sq., Dyenamo), propylene carbonate (PC, 99.5%, Thermo Scientific), acetone (99.8%, Acros Organics), toluene (99.85%, Acros Organics), DMSO-d_6 (99.9 atom %, Sigma Aldrich), ethylene carbonate (99.92%, *Trace***CERT**®, Sigma Aldrich), toluene-d_8 (99.5%, Deutero), tetrabutylammonium hexafluorophosphate (TBAHFP, 98 %, AlfaAesar), ferrocene (Fc, 98 %, Acros Organics), tetrabutylammonium bromide (TBAmBr, ≥99%, Sigma Aldrich).

$CsPbBr_3$ Nanocrystal Synthesis according to Protesescu et al.[1]
163 mg (0.5 mmol) of Cs_2CO_3, 0.5 ml (1.6 mmol) of OA and 8 ml of ODE were loaded into a three-neck flask and dried at 100 °C for one hour. Afterwards, the solution was heated up to 150 °C for 10 minutes in N_2 atmosphere to form Cs(oleate). In a second flask, 422 mg (1.15 mmol) $PbBr_2$ were added to 30 ml ODE and degassed at 100 °C for one hour. 3 ml (9.45 mmol) of OA and 3 ml (9.12 mmol) of OAm are added under N_2. Again, the flask was heated up to 100 °C and degassed for half an hour. Afterwards, the temperature was elevated to 165 °C and 2.4 ml of Cs(oleate) were injected rapidly. The solution was cooled down to room temperature after 5 s by an ice-bath. The crude solution was centrifuged at 8000 rpm for 20 minutes and the precipitate was resuspended in toluene. Agglomerates were removed by slow centrifugation at 2500 rpm for 10 minutes. The supernatant passed a syringe filter and was characterized by fluorescence and absorbance spectroscopy in solution at a PerkinElmer FL8500 spectrometer and Agilent Technologies Cary 5000 UV/Vis spectrometer, respectively. The size of the NCs was determined by scanning electron microscopy. SEM images were recorded at a Hitachi SU8030.

NC Preprocessing.
The partial ligand exchange (ideal: 141 lecithin/NC) was performed in solution by adding lecithin in toluene (0.75 mM, 434 µl) to the NC stock solution (7.6 µM, 300 µl) and stirring for 30 minutes. Afterwards, the solution was precipitated by adding twice the volume of acetone as anti-solvent. The partially ligand exchanged NCs were resuspended in toluene again.

Self-assembly into SCs.
Before-self-assembly, the FTO substrates were cleaned by ultrasonication for 15 mins in each acetone, Extran® (Merck Millipore), and distilled water. For crystallization, the substrates were placed in a petri dish as crystallization chamber in a nitrogen filled glovebox. 150 µl of a 0.25-0.4 µM solution of NCs in toluene were drop-cast on the tilted FTO substrate (angle 10-20 °). Additionally, a solvent reservoir filled with toluene was kept in the crystallization chamber to create an oversaturated solvent atmosphere. The solvent was left to slowly evaporate for 3 to 5 days. The SCs were identified at a light microscope.

Quantitative NMR sample preparation.

A specific amount of NCs (300 µl) of a 3 µM solution was used for quantitative NMR spectroscopy. Here, the solution was dried and dissolved in 0.5 ml of a 15 mM ethylene carbonate DMSO-d_6 solution. 64 scans of protons were recorded with a relaxation time of 40 s. The spectra were recorded at a 400 MHz BrukerAvance400 III HD.

SEC PL setup.

The spectroelectrochemical cell is home-built. It consists of an Ag wire as pseudo-reference electrode, a Pt coil as counter electrode, and a transparent FTO is used as working electrode. The cell was filled with 5 ml of a 0.1 M solution of PC/TBAHFP. TBAHFP was recrystallized five times from EtOH/H_2O and dried for five days under vacuum at 105 °C., beforehand.

The cell containing the self-assembled NCs on FTO and filled with the ES was placed on top of an inverted confocal microscope. Then, the electrodes were connected to a CHI Instruments potentiostat (CHI760E), the open circuit potential was measured, and *iR* compensation was applied.

High-resolution optical characterization.

Spectral mapping was performed using a home-built inverted confocal laser piezostage-scanning microscope. A 405 nm laser diode (LDH P-C-405, Picoquant GmbH), operated in continuous wave mode, was used as the excitation source. Focussing of the excitation light and collecting scattered and emitted light was achieved using an infinity corrected, long working distance 50x/0.6 numerical aperture air objective (Nikon). A 458 nm long-pass filter (RazorEdge LP Edge Filter 458 RU, Semrock) was inserted in the detection path to remove the excitation wavelength. Photoluminescence spectra were recorded by a DU4A01-BVF camera (Andor/Oxford Instruments) cooled to -60 °C attached to an SR-303i-B spectrometer (Andor/Oxford Instruments) using a 300 grooves/mm grating. Scanning of the piezo stage and interfacing with the spectrometer for spectral mapping was achieved by the HydraLabX1 controller (HydraSpex UG).

S2. Qualitative and quantitative ^{1}H NMR spectroscopy.

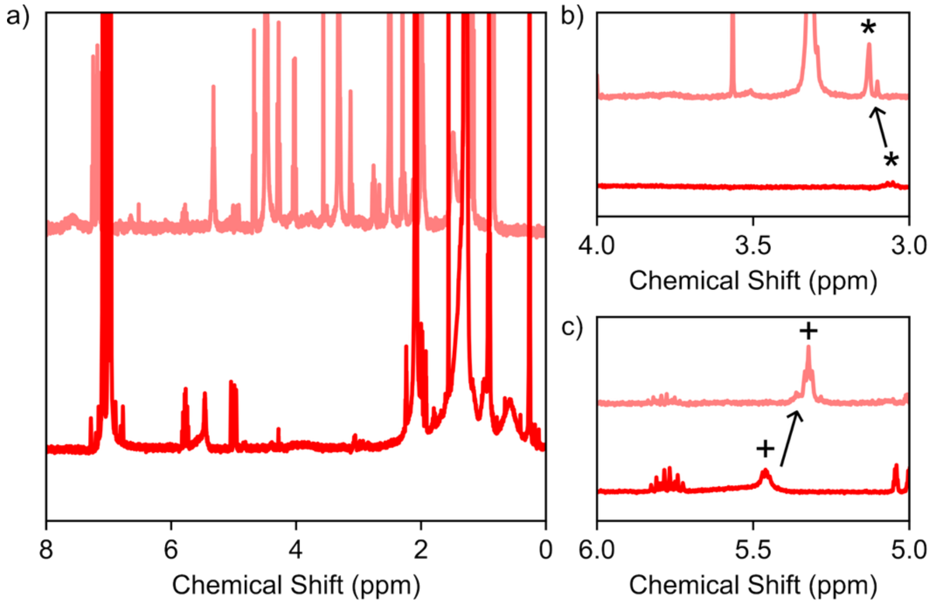

Figure S1. ^{1}H NMR of exchanged NCs, top (light-red): quantitative NMR with ethylene carbonate as standard (4.5 ppm) in DMSO-d_6 (2.5 ppm), bottom (dark-red): NC NMR in toluene-d_8. **a)** full spectra, **b)** zoom-in between 4 and 3 ppm, highlighting methylgroups of the phosphocholin group (*), and **c)** zoom-in at vinyl protons of OA/OAm (+).

Calculation of ligands/NCs from quantitative NMR spectroscopy.
n(Ethylene Carbonate) = 2.25×10^{-5} mol
n(max. NCs in qNMR, used for ligand exchange) = 1.14×10^{-9}mol
purity (lecithin) = 97 %
purity (OA/OAm) = 90 %
purity (ethylene carbonate) = 99.92 %

	Integral	n(ligands)	ligands/NC
Vinylpeak (2 Hs, OA/OAm)	0.0511	6.4×10^{-7} mol	561
Methylgroups (9 Hs, lecithin)	0.0224	5.7×10^{-8} mol	50

S3. SEM characterization of NCs before and after ligand exchange (without crystallization).

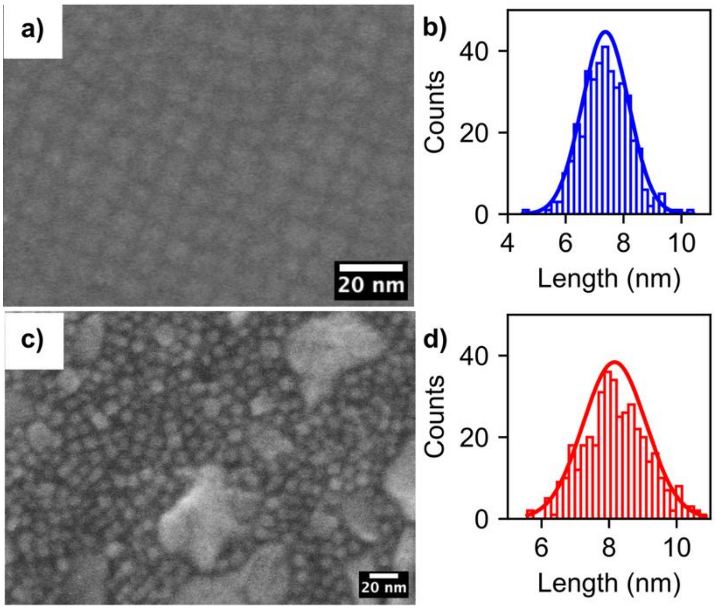

Figure S2. SEM characterization of NCs before (top) and after (bottom) ligand exchange. a) NCs spin-coated on a silicon substrate and determined average size of nm 7.4 +/- 0.8 nm by fitting to normal distribution from SEM image, b) NCs spin-coated on FTO substrate and determined average size of 8.2 +/- 0.9 nm by fitting to normal distribution.

S4. SC characterization.

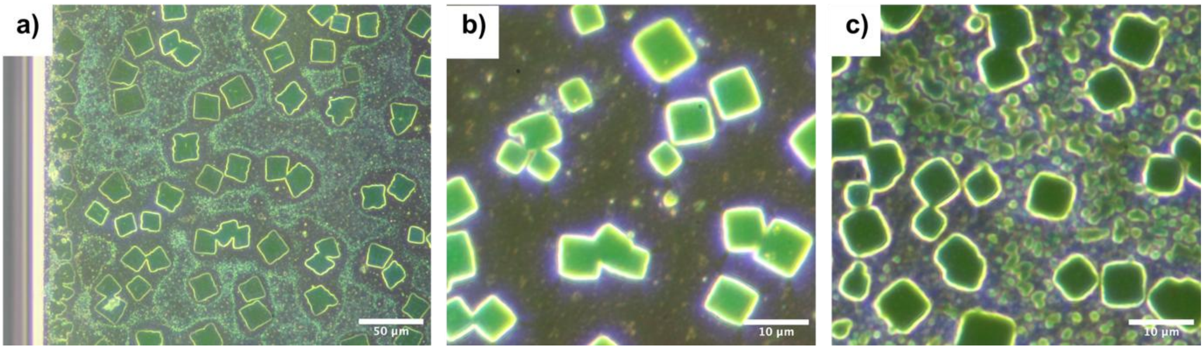

Figure S3. Light microscopy in the dark-field mode on three different samples with SCs.

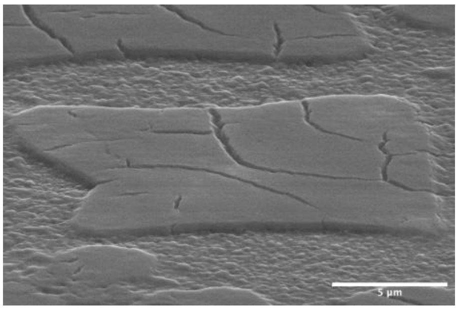

Figure S4. SEM of tilted SCs on FTO showing thickness of approx. 0.5 μm for the SC and distribution of NCs over the whole sample.

S5. SEC PL cell and referencing by the Fc/Fc⁺ redox couple.

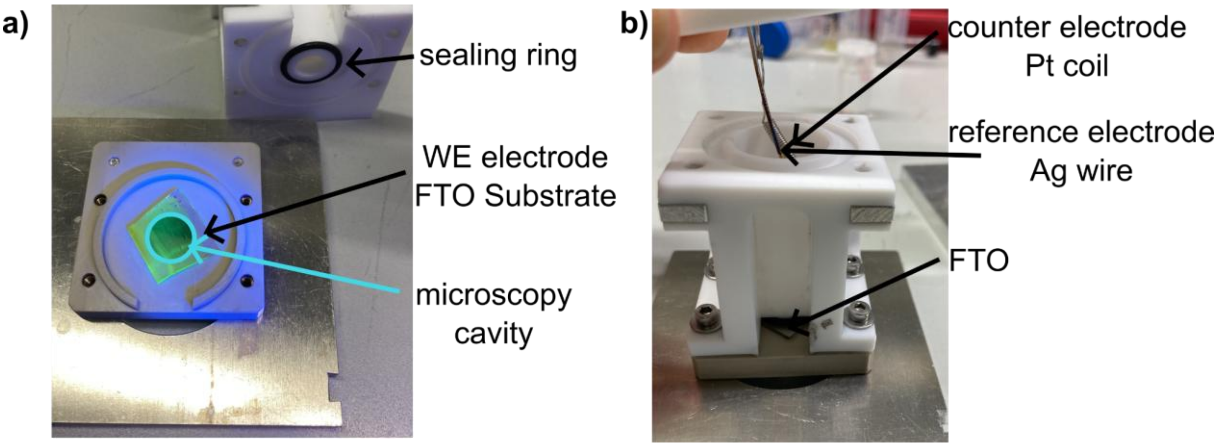

Figure S5. Photos of the SEC PL cell. a) bottom layer with exemplary SCs on a FTO substrate and b) assembled SEC cell for measurements.

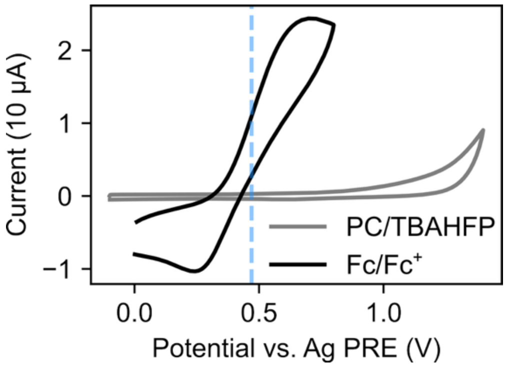

Figure S6. Cyclic voltammetry (CV) with a scan speed of 50 mV/s of electrolyte solution (PC/TBAHFP, gray) and Fc/Fc^+ redox couple (black) with determination of the half-wave potential (dashed blue line) at $E_{1/2}(Fc/Fc^+)$ = 0.47 V vs. Ag PRE.

S6. PL spectra extracted from the spectral maps in Figure 2b+c (main mansucript).

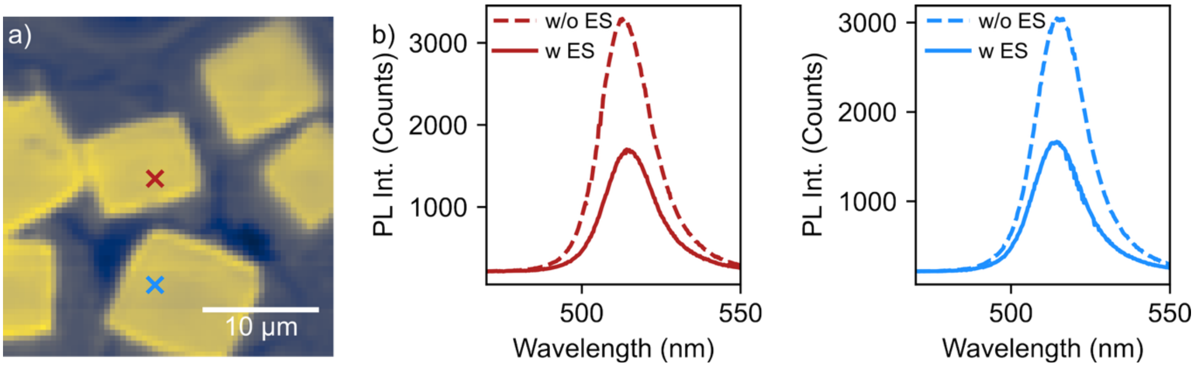

Figure S7. Comparison of PL spectra at two positions without and with added electrolyte solution. a) integrated intensity map for positioning of extracted spectra, b) comparison of PL spectra of position 1 with electrolyte solution (ES, solid line) and without ES (dashed line), and c) PL spectra comparison of second position.

The PL decays by 49 and 47 % for position 1 and 2, respectively.

S7. SEC PL including bromide oxidation from solution.

In the following experiments, the SCV window was extended up to 0.98 V. In this electrochemical window, the oxidation of free bromide occurs. This oxidation leads to a complete dissolution of the SC, resulting in a rapid decay of the PL. The irreversible PL decay starts at 0.78 V as shown in **Figure S7b**. For further confirmation of the bromide oxidation, CV of tetrabutylammonium bromide (TBAmBr) in solution (**Figure S8**) was conducted (in the SEC PL cell). This CV shows the coinciding bromide oxidation at 0.78 V. For the conducted experiments in **Figure 3**, the consequence is that bromide oxidation is also occurring, but the potential and time window is sufficiently limited, so that the SCs are not decomposing within the measurement.

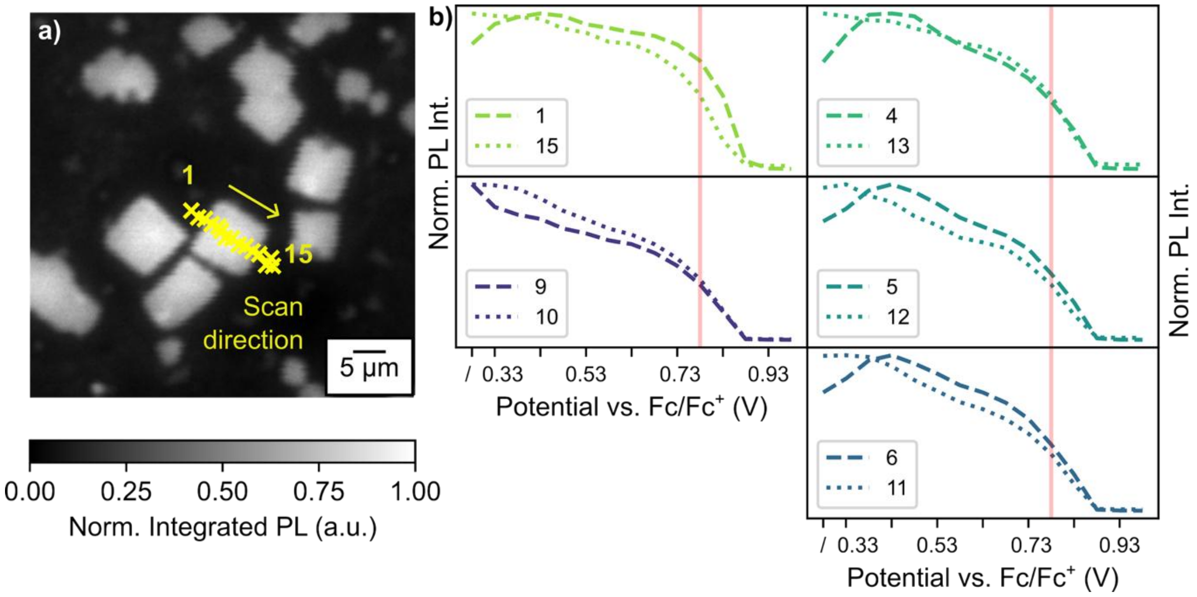

Figure S8. SEC PL on SC from 0.33-0.98 V. **a)** Intensity map of SC with chosen positions for probing PL intensity during the application of a potential. **b)** PL intensity trace for all positions with bromide oxidation resulting in complete dissolution of SCs.

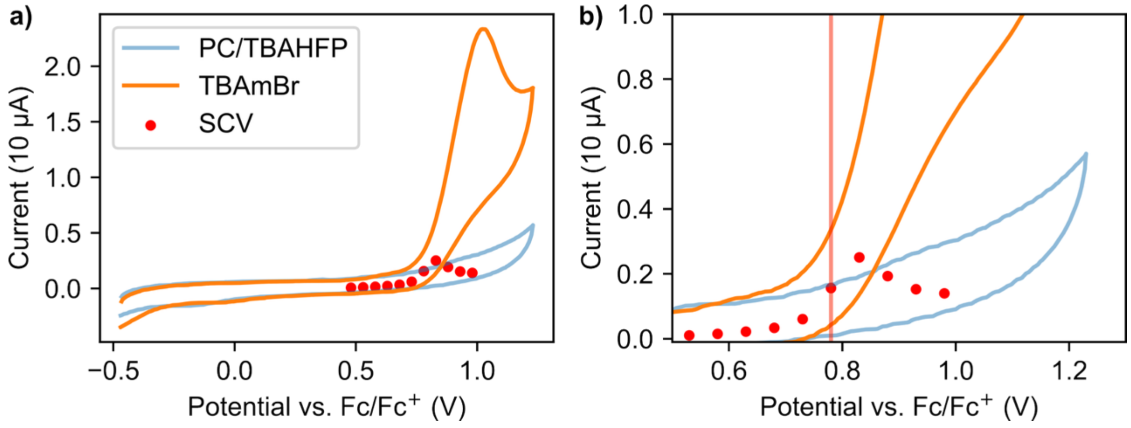

Figure S9. Electrochemical data recorded during SCV of SC (red dots, corresponding to **Figure S8**), CV of electrolyte solution (bright blue), and CV of TBAmBr in PC/TBAHFP (orange). Scan speed of CVs was set to 50 mV/s. **a)** Full CV plot, and **b)** zoomed-in CV plot.

S8. Evaluation of SEC PL in Figure 3.

In **Figure S10**, all spectra recorded at each potential step are shown. The first spectrum without the applied potential is the same as in **Figure 3b** without normalization. These spectra were used for calculating the differential PL in **Figure 3d.**

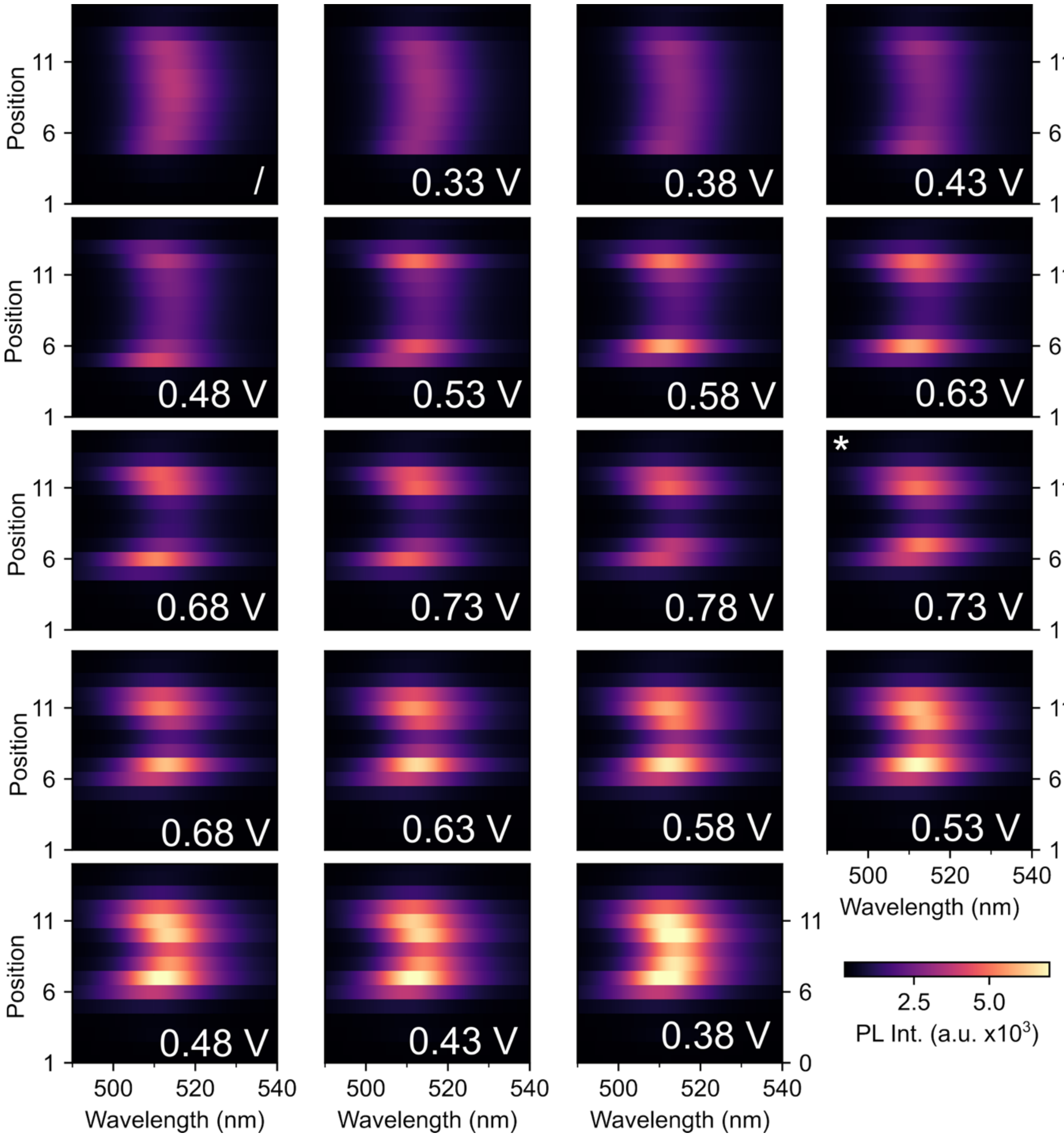

Figure S10. Single spectra recorded at each position and each potential (going from top left to bottom right). The asterisk highlights the first spectra recorded in the reversed scan direction.

More details can be gained when the PL intensity is plotted against the applied potential for specific positions. As apparent in **Figure 3**, the PL behavior changes going from the edges to the center. Hence, we have chosen to investigate the PL intensity of both of the edges (light blue) going from position 5 and 6 into the center (dark blue) of the SC and from 13 to 11 as depicted in **Figure S11**. In **Figure S11a**, we observe that the increase in PL intensity starts with position 5 at lower potentials (0.38 V), while the PL increases

drastically above 0.48 V for position 6. The shift in the onset potential of PL brightening can be also observed on the other edge going into the SC center in **Figure S11b**. The onset potential for position 13 and 12 is at 0.43 V, while for position 11, it is shifted to a higher potential (0.53 V).

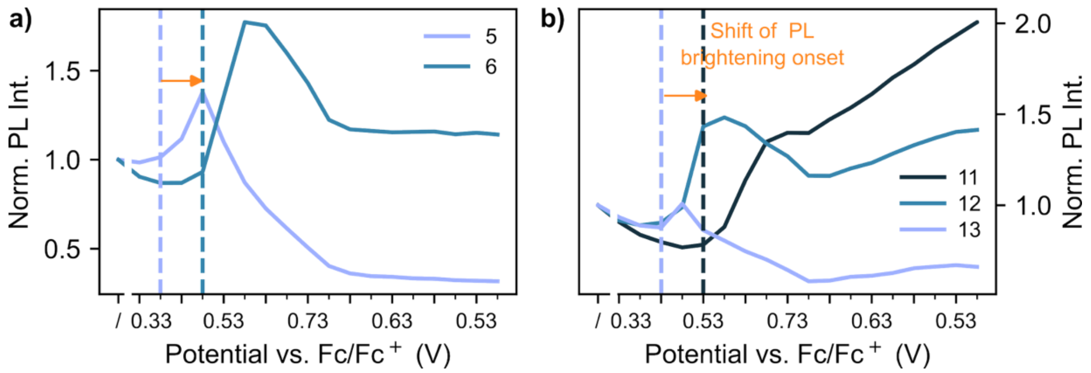

Figure S11. Plot of maximum PL intensity normalized to the maximum PL intensity without applied potential PL_0 with dashed lines indicating the onset potential of PL brightening **a)** for the right edge of the SC of positions 5 and 6, and **b)** for the left edge of the SC of positions 11, 12, and 13.

The center positions behave differently than the edge positions. Here, the PL increase can only be observed, when the maximum applied potential is surpassed and the scan direction is reversed.

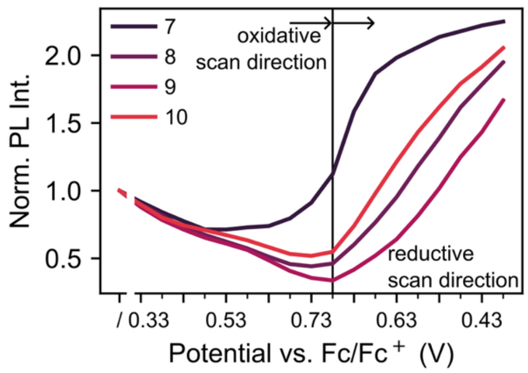

Figure S12. Plot of the peak PL intensity normalized to the peak PL intensity without applied potential PL_0 for the center positions of the SC 7, 8, 9, and 10 with the solid line at the maximum applied potential (0.78 V).

S9. Determination of the position-dependent hole injection potential in a SC.

In another measurement, we performed a SCV scan up to 0.63 V vs. Fc/Fc⁺, thereby avoiding the start of Br⁻ oxidation as it can be observed by the low current in the SCV in **Figure S13c**. The positions were again probed as in the main manuscript in **Figure 4**.

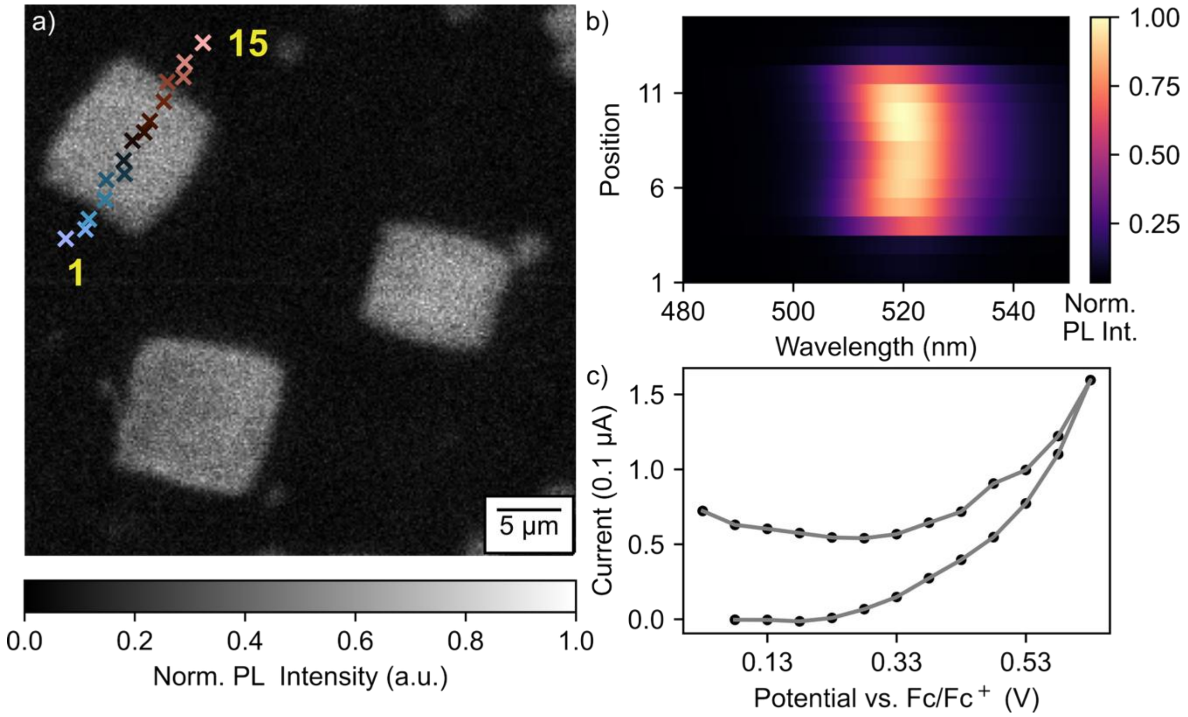

Figure S13. Spectral mapping of positions within a SC. a) PL intensity map of three SCs with the positions indicated on the probed SC, b) normalized PL spectra before SEC recording at specific positions, and c) SCV diagram.

In the next two figures, the PL intensity normalized to PL_0 is shown. The PL lines are split into upper and lower edge positions, going from the edge (light blue) to the SC center (dark blue). In the figures, the onset potential of PL decay is indicated by a dashed line, defined as the potential after which the PL intensity decays by > 5 % on the next step.

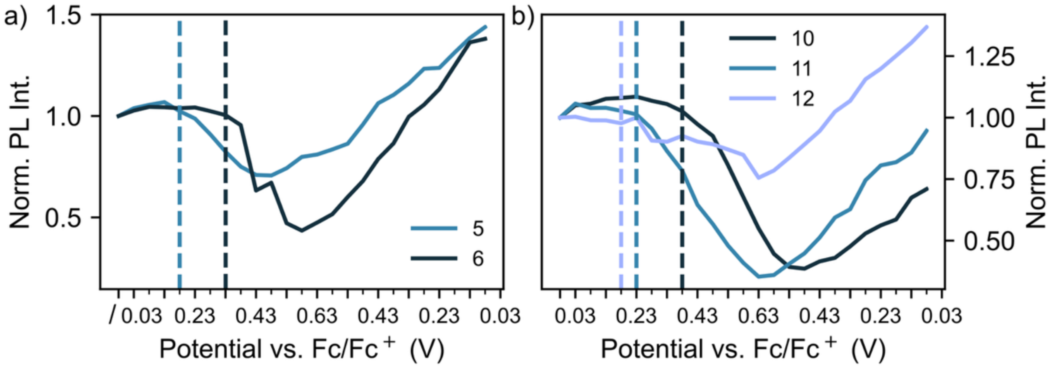

Figure S14. PL intensity trace with indicated onset potential of PL decay (dashed line) depending on the applied potential for a) position 5 and 6, and b) position 10, 11, and 12.

In **Figure S15**, we observe a PL decay in the SC center starting at 0.33-0.43 V. The PL starts increasing again when the scan direction is reversed. For position 7 and 8, we observe a full recovery of the PL or even a PL enhancement (for position 7). The PL intensity behavior

of darkening at a specific potential and PL recovery in the reversed scan can be attributed to a reversible electrochemical hole injection.

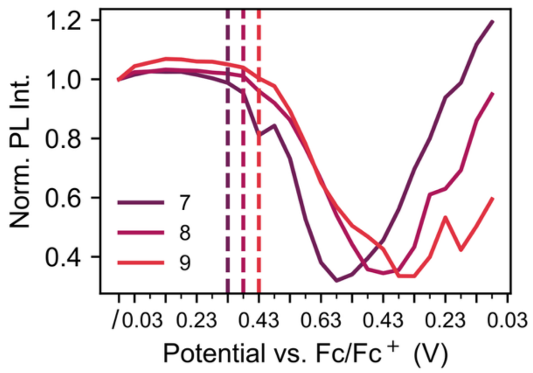

Figure S15. PL intensity trace with indicated onset potential of PL decay (dashed line) depending on the applied potential for the center positions of the SC.

The onset potentials of PL decays at different positions are summarized in **Figure S16**. The data highlights the shift of the onset potential to higher values in the SC center compared to the edges.

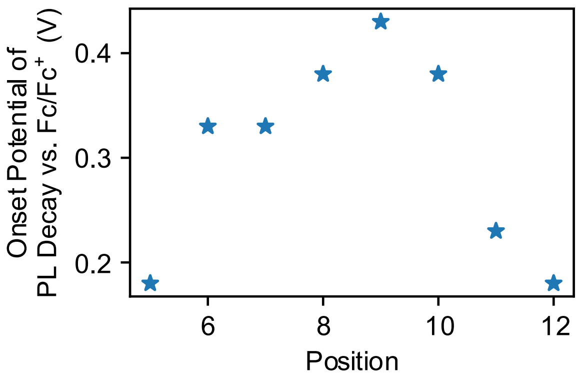

Figure S16. Summary of onset potentials of PL decay depending on applied potential for the positions in the SC.

S10. Absorbance SEC on NC thin-film.

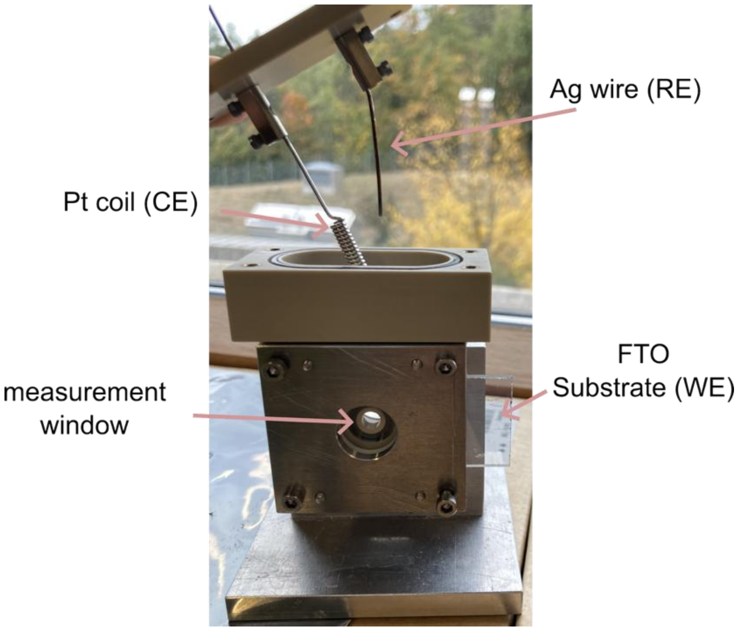

Figure S17. Photograph of the spectroelectrochemical cell for absorbance measurements.

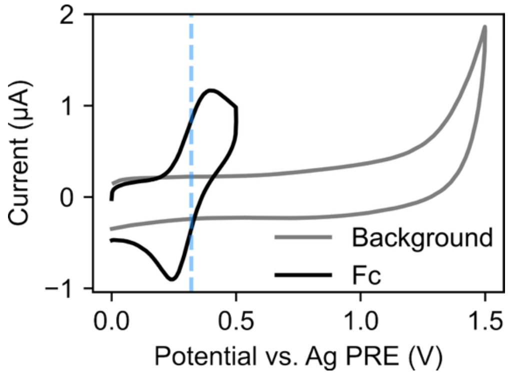

Figure S18. Reference measurement of Fc/Fc^{+} redox couple in PC/TBAHFP in the SEC absorbance cell as depicted in **Figure S17** with determined half-wave potential $E_{1/2}$= 0.32 V vs. Ag PRE (blue dashed line).

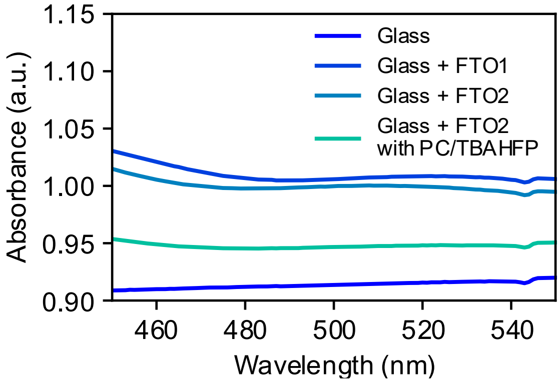

Figure S19. Absorbance of the spectroelectrochemical cell used without any NCs drop-cast on the FTO WE.

SEC in absorbance was recorded on a NC thin-film since there is no optical resolution for these measurements to find specific SCs. We did apply the same SCV recording parameters as in **Figure 3**. Additionally, absorbance spectra of only added electrolyte solution and solvent were recorded in the same time window as the SCV spectra were recorded (1h 10 minutes, first spectrum dark, last recorded spectrum bright green).

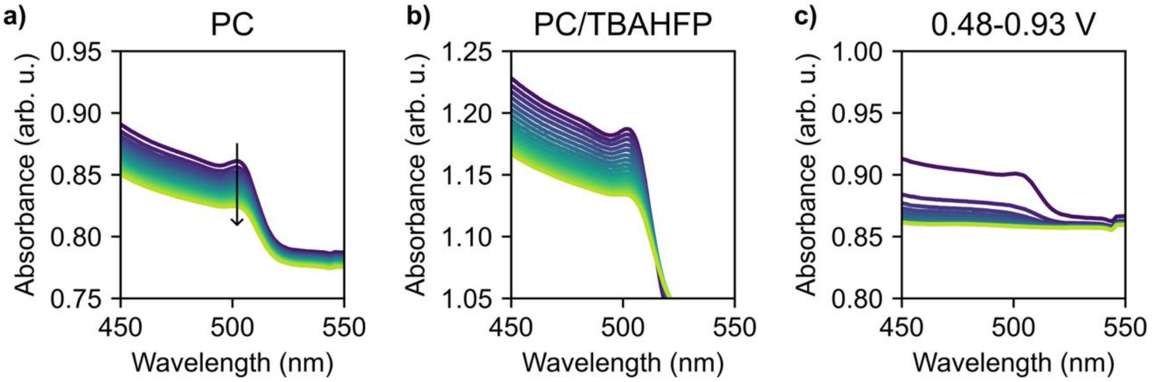

Figure S20. Absorbance spectra of a NC film over 1h 10 minutes. **a)** Only with added solvent propylene carbonate, **b)** with electrolyte solution (PC/TBAHFP), and **c)** with SCV scan from 0.48 – 0.93 V in 50 mV increments, each potential applied for 210 s.

SEC in absorbance was also recorded at different applied potentials w/o, -0.12 V, 0.18 V, and 0.68 V (keeping one potential) for 10 spectra, and then stopping and letting the absorbance recover.

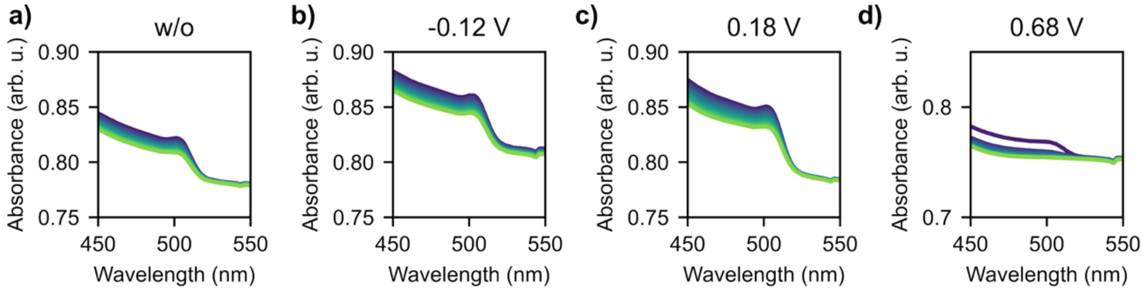

Figure S21. Potential-dependent absorbance of NC film a) without applied potential (with PC/TBAHFP), b) at -0.12 V, c) at 0.18 V, and d) at 0.68 V vs. Fc/Fc^{+}.

The absorbance measurements conducted in PC, PC/TBAHFP and at the potentials of -0.12 and 0.18 V show a gradual decrease of the first excitonic transition. The gradual decrease in absorbance can be attributed to the instability of the NC thin-film in PC since the gradual decrease is recorded already for the NCs only covered by PC in **Figure S20a**. In **Figure S20c** and **S21d**, an additional absorbance bleach can be detected in the second recorded spectrum. The absorbance bleach in **Figure S21c** can be assigned to the applied potential of 0.53 V. The constant applied potential of 0.68 V in **Figure S21d** is higher than 0.53 V and results in a decay of absorbance. In this plot, the first excitonic maximum of the first recorded spectrum is not as pronounced as in **Figure S20c**, which is already indicating that the absorbance bleach is already recorded in the first spectrum. Both SEC in absorbance measurements show the absorbance bleach at a higher potential than 0.53 V, which is now considered as the hole injection potential.

S11. Spectral mapping of another SC example.

The same SEC PL parameters were applied to another sample of SCs on a FTO WE. Before and after SEC PL, spectral maps were recorded. The spectral maps (**Figure S22a+b**) show PL brightening of approx. 100 % of integrated PL intensity of the SC after conducting SCV. Only a shift of 1 nm in maximum peak wavelength can be observed for extracted single spectra in the SC (**Figure S22c**).

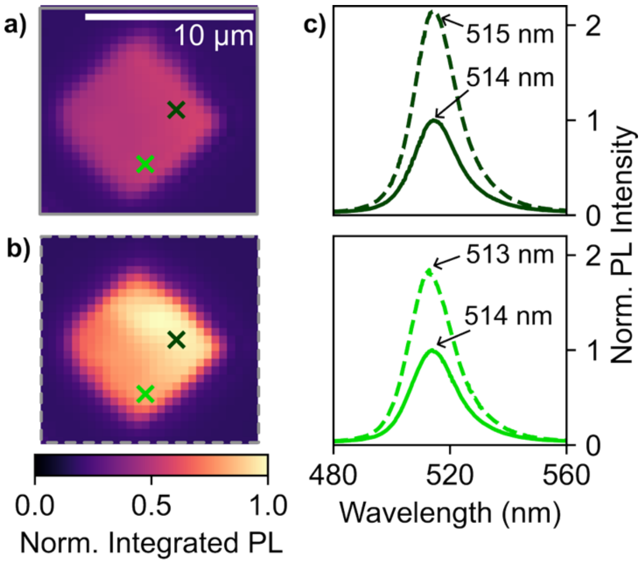

Figure S22. Spectral map before (top) and after (bottom) application of SCV in a potential range of 0.33-0.78 V vs. Fc/Fc^{+}. **a)** Normalized integrated PL intensity of SCs before applied potential, with added electrolyte solution. **b)** Normalized integrated PL intensity after SCV. **c)** Extracted single spectra from recorded spectral maps before (solid line) and after SCV (dashed line) normalized to PL intensity before SCV.

S12. Time-resolved PL after electrochemical hole injection.

We performed time-resolved PL measurements on SC samples before and after SCV. For the measurement before SCV, we summed up the PL decays of the SCs shown in **Figure S24**. The histograms after SCV were recorded at specific positions in the SCs. Two positions show only moderate brightening (blue and red), while the third position shows strong brightening. After fitting the decays to a biexponential fit function, we determine the lifetimes of the fast and slow component. We find a decrease from 1.7 to 0.7 ns in the short lifetime (τ_1) for the dim SCs, while τ_2 does not change significantly (7.7 to 6.9 ns). In contrast, the strongly brightened position (green) exhibits a prolonged τ_2 component (15 ns vs. 7.7 ns), while τ_1 is comparable to the one of the SCs before SCV (1.7 to 1.6 ns).

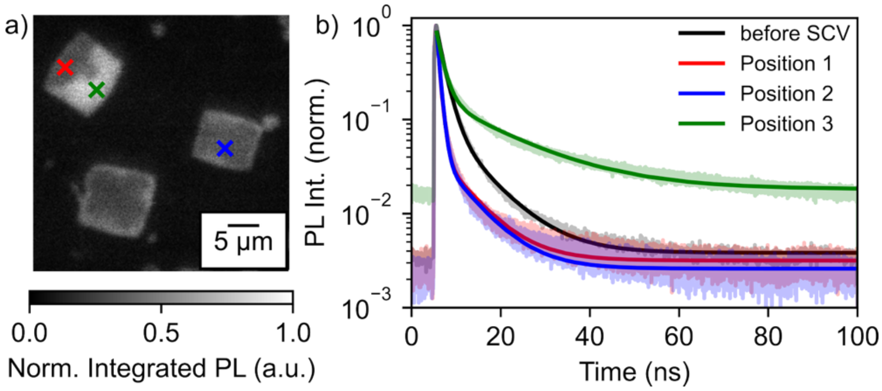

Figure S23. Time-resolved PL of SC at different positions on the SC sample. a) PL intensity map and b) PL decays and their biexponential fit, recorded by means of time-correlated single photon counting for the positions indicated in a) compared to PL decay before SCV in black.

Biexponential fit function:

$$I(t) = A_{rel,1} \exp\left(-\frac{t}{\tau_1}\right) + A_{rel,2} \exp\left(-\frac{t}{\tau_2}\right)$$

Table S1. Fitting parameters extracted from the biexponential fits of PL decays in **Figure S23b**.

Position	$A_{rel,1}$	τ_1 (ns)	$A_{rel,2}$	τ_2 (ns)	$\tau_{Av, Amp}$ (ns)	$\tau_{Av, Int}$ (ns)
Before SCV - black	0.8894	1.71	0.1106	7.7	2.37	3.87
1 – red	0.94628	0.7234	0.0537	6.91	1.056	2.9
2 – blue	0.9488	0.734	0.0512	6.89	1.05	2.8
3 - green	0.8223	1.56	0.177	15	4	11

S13. Statistics on PL enhancement after conducting a SCV scan.

In the following, two spectral maps with a larger sample area were recorded. A SCV scan in the potential range of 0.33-0.73 V was conducted, comparable to the SCV scan in the main manuscript. During that time, the sample was not exposed to laser irradiation. Afterwards, the SCs show a higher PL intensity while the NC film starts degrading (lower edge of **Figure S24b**).

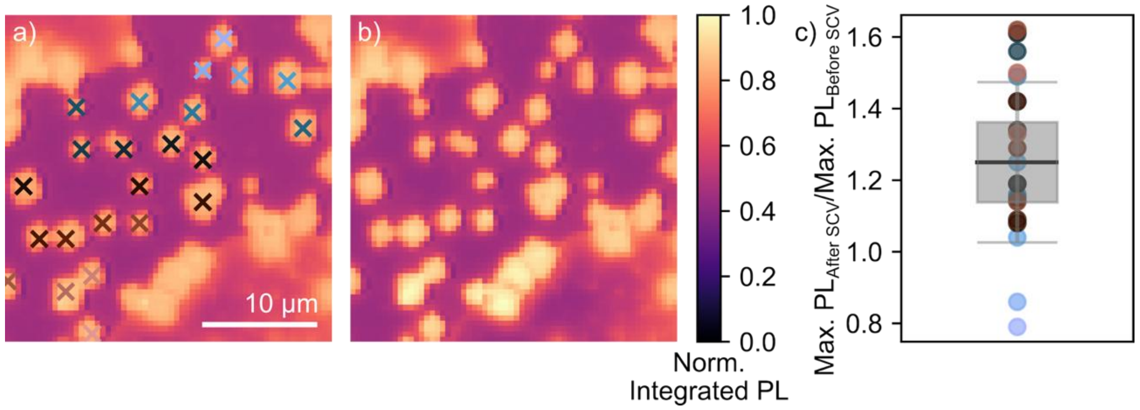

Figure S24. Spectral comparison of spectral maps before and after a SCV scan with many SCs. **a)** Integrated PL intensity map before SCV, **b)** integrated intensity map after SCV, and **c)** change in PL intensity after SCV scan of 22 SCs, indicated in S24a.

Summarizing, we find in the spectral map 22 evaluated SCs, 20 of which (91%) exhibit significant PL enhancement. The intensity change is 1.26 ± 0.22.

S14. Effect of edge brightening.

After conducting a SCV scan in the potential range of 0.03-0.63 V, the comparison of the PL intensity maps before and after SCV show PL brightening and edge brightening of all recorded SCs in the probed region. For SC2 and SC3, the non-probed SCs during SCV, we find PL 1.4 and 1.7-fold PL brightening, respectively.

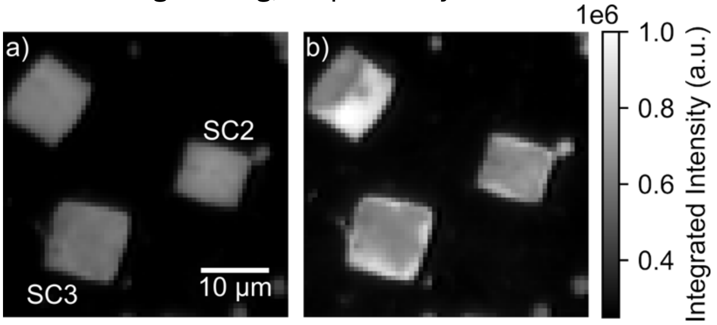

Figure S25. PL intensity map of SCs before (panel **a**) and after SCV scan (panel **b**) from 0.03 up to 0.63 V.

References

(1) Protesescu, L.; Yakunin, S.; Bodnarchuk, M. I.; Krieg, F.; Caputo, R.; Hendon, C. H.; Yang, R. X.; Walsh, A.; Kovalenko, M. V. Nanocrystals of Cesium Lead Halide Perovskites ($CsPbX_3$, X = Cl, Br, and I): Novel Optoelectronic Materials Showing Bright Emission with Wide Color Gamut. *Nano Lett.* **2015**, *15* (6), 3692–3696. https://doi.org/10.1021/nl5048779.
